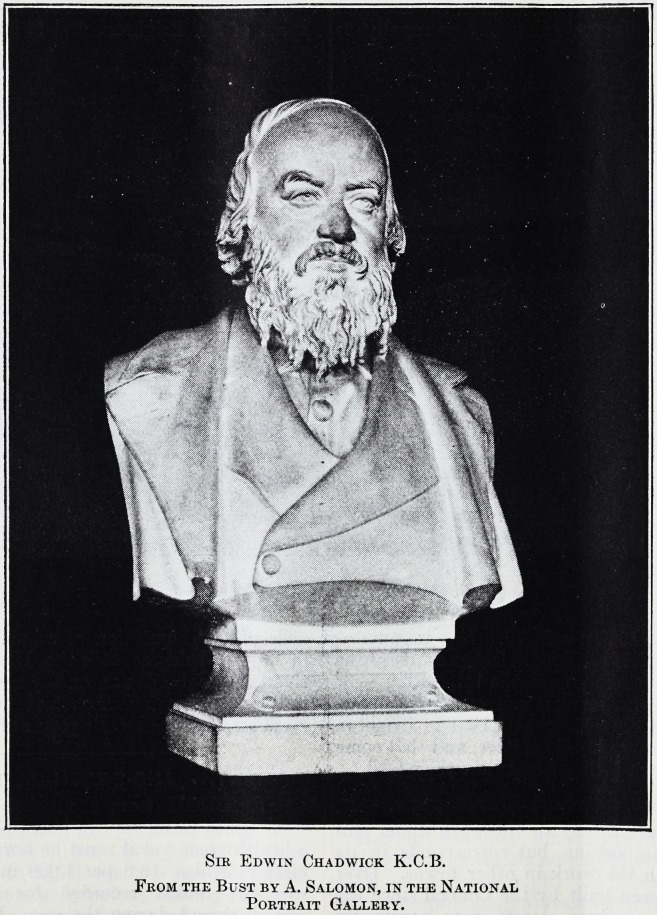# The Life and Doctrine of Sir Edwin Chadwick

**Published:** 1924-02

**Authors:** William J. Collins


### THE LIFE AND DOCTRINE OF SIR EDWIN CHADWICK.

By SIR WILLIAM J. COLLINS, K.C.V.O., M.D.

THE following is the substance of a lecture upon the life and work of Sir Edwin Chadwick delivered recently by Sir William J. Collins, K.C.V.O., M.D., at University College, London.

### A Great Victorian.

Chadwick belonged to the noble army of eminent Victorians whose times and tendencies it is now the fashion to belittle and besmirch, though his early activities in association with Bentham, Grote and the Mills, father and son, commenced some years before the accession of Queen Victoria. When that reign began, as Sir John Simon truly observes, "there existed hardly a glimmer of intelligent public interest in matters affecting the public health." The changes that came in quick succession during the following decade (1838–1848), were, according to the same eloquent chronicler of English Sanitary Institutions, "directly due to the zealous labours of one eminent public servant, Sir Edwin Chadwick."

Sir Edwin Chadwick K.C.B.
From the Bust by A. Salomon, in the National Portrait Gallery.

### A Message for To-day.

Apart from minor matters, mostly of ephemeral interest, Chadwick's teaching is not without its message for us to-day. Some, at any rate, of our modern sanitarians and sociologists seem to lack the very qualities in which Chadwick was preeminent. They have not the massive grasp, the many-sided method of approach, the hold on totality which he so conspicuously possessed. Even Sir Benjamin Ward Richardson, who had so much in common with his hero, alike in heart and head, found Chadwick's personality defiant of ordinary classification and not easy to analyse. It was fortunate, indeed, that in the evening of his long life Chadwick found in Richardson, my old friend and colleague, a biographer after his own heart, who under his hero's eye made a worthy record of his varied beneficent activities. In 1887 Richardson published in two volumes "The Health of Nations: a review of the work of Edwin Chadwick with a biographical dissertation." Articles, reviews, reports, memoranda, blue-books flowed in rapid succession for threescore years from the facile pen of Edwin Chadwick, and it is well to take down, dust and reperuse them, and to reflect on them in the light of twentieth-century sanitary and social science. No one can rise from that task without a vivid impression of their sanity of judgment, their modernity and presage, their large and liberal outlook, their insight and foresight, as well as of the breadth of view and the humanity of the philosophy which inspires them.

### Chadwick as Journalist.

Chadwick's long life of ninety years spanned the nineteenth century with the exception of its concluding decade. He and the century were in their teens together, for he was born at Longsight, near Manchester, on January 24, 1800. His times, upbringing and early associations combined to mould this Lancashire lad into the indomitable and indefatigable social and sanitary reformer for which occasion was so ripe. Before he was ten years old he migrated with his father to London, whither the latter had been called to fill the editorial chair of the *Statesman*, a leading Liberal paper of that time. In London, young Chadwick devoted himself to the study of modern languages, he attended no public school except preparatory ones at Longsight and Stockport, and entered for the Bar at the Inner Temple; but with his father's example before him, journalism claimed his early attention. Articles from Chadwick's pen on "Life assurance" and "Public charities in France" stressed the potency of environment on health and exhibited in embryo his "Sanitary idea," which afterwards germinated and developed. An essay on "Preventive Police" attracted especially the attention of Bentham, secured his patronage and friendship and introduced him into the brilliant circle of the devoted disciples who surrounded the utilitarian philosopher in his closing years.

### The "Sanitary Idea."

Bentham, who was born in 1748, three years after the death of Walpole, died the day before the Great Reform Bill of 1832 received the Royal Assent. In the evening of his life Chadwick and Dr. Southwood Smith resided with him at his hermitage at Queen's Gate, Westminster, and acted as secretaries during the last three years of the octogenarian sage. Besides a share of his library, the master bequeathed a legacy to Chadwick, who had no doubt acquired from Bentham a breadth of view and basic notions of a juridical system which powerfully fomented within him the forces of his developing personality. Before he was thirty, he had already become possessed of, or rather, possessed by, his "Sanitary idea." He proceeded to study typhus fever amid the rookeries of East London, and nearly fell a victim to it. He became intimate with John Stuart Mill, and, it is said, laid unsuccessful siege to the heart and hand of his sister Harriet. Mill introduced him to Nassau William Senior, and through him, in 1832, he received the appointment of Assistant Commissioner for the enquiry into the Poor Law which Lord Grey's Government had just resolved to initiate under the Chairmanship of the learned and large-hearted Bishop Blomfield. From the vantage ground of this relatively subordinate position, Chadwick was started on what was destined to be his life's work.

### Reforming the Poor Law.

The Commission recommended the formation of a Central Board to control administration and to prescribe uniform regulations as to relief while local unions of parishes were to provide workhouses, administer relief, educate and apprentice children, levy rates, appoint paid officers, and render uniform accounts, under central supervision. Relief to able-bodied paupers was to be declared illegal. The law of settlement was to be relaxed. The first three Commissioners, with Chadwick as their most energetic Secretary at once proceeded by rules and orders to regulate outdoor relief, to make proper classification of the infirm, the aged, the destitute, the lunatic, the children, and the vagrant, and prescribe their respective appropriate treatment—in modern phraseology, "to break up the poor law."

### Juvenile Labour.

Acute internal dissensions rent the Board. A Committee of which Disraeli was a member, was appointed to inquire into the affair by the Commons, against the wish of the Government, and reported in vindication of Chadwick and against the Commissioners, who accordingly resigned in 1847. Other work was, nevertheless, soon found for his indomitable energy. Even while working on the report of the Poor Law Commission and for the Board as Secretary he had undertaken other investigations. In 1833 the grim tragedy of juvenile labour in factories had, by a single vote in the Commons, been referred to a Commission. Thomas Tooke, Southwood Smith and Chadwick were the Commission, and with alacrity they enquired and reported. Legislation, however, lingered since the Lords resented what they described as an insidious attempt to introduce by a sidewind universal national education. The half-time system, of which Chadwick was the proud parent, then ten-hours day, the institution of Government factory inspectors and the recognition that injuries of employees were a legitimate charge on an industry, may be regarded as the early or the later fruit of the Commission of 1833.

### Government Neglect of Health.

Modern students of public health would find it difficult to visualise the social misery and mal-hygiene which prevailed among the masses of the people at that date. The first great cholera epidemic which struck Great Britain in 1831 had caused a rude awakening and served to draw attention to the conditions under which the poor lived and died in large urban communities. It was not, however, until the Chadwick régime that the potency of filth in air, in water, food, soil and surroundings became recognised in preparing the breeding ground for epidemics, and sanitary neglect began to be denounced as the nurse, if not the parent, of contagious fevers. Chadwick's monumental *Survey into the Sanitary Condition of the Labouring Classes of Great Britain* was the final blow to the "letting-things-alone" party in high places, and led to the institution of local Medical Officers of Health.

### Laying the Foundations.

He believed in the common origin of zymotics in sanitary neglect—"that smallpox follows on much the same lines as typhus, so does scarlatina, but with wider deviations as to classes of cases and conditions of persons." The propagation of fever he traced largely to untrapped drains; and the use of efficient traps and glazed earthenware self-cleansing pipes was early advocated by Chadwick. He urged the prompt isolation of infected persons in a well-ventilated chamber, so as to "let a current of air pass through the room and over the patient." He enunciated precise rules for the sanitary construction of dwellings and hospitals, laying special stress on ample cubic space to get rid of "vitiated, phthisisproducing air, and (if the crowding is intense) fever-producing air."

### Fall of the Central Board.

The Public Health Act of 1848 was adoptive only, did not apply to London, and was limited in duration (unless renewed) to a period of five years, nor did it give effect to all that Chadwick desired. It nevertheless, for the time being, stabilised and strengthened the General Board of Health with the Earl of Shaftesbury (at that time Lord Ashley) as unpaid Chairman and Chadwick, and later Southwood Smith as its paid members. It was efficient, economical, assiduous. It applied strong action and stimuli from headquarters to lethargic local authorities and, proceeding by Provisional Orders rather than by Private Bills, it gave umbrage to parliamentary agents whose emoluments were thereby reduced. The cry of " Centralisation " was raised, the dictatorial edicts and *doctrinaire* policy of the Board were deronuced by the " practical men " who were content that sleeping dogs should be permitted to lie and sanitary neglect continue as ever of old. Matters came to a crisis ir the summer of 1854 when all the disaffected interests became vocal in the Commons, and on July 31 a combined attack on the proposal to renew the Act of 1848, with its General Board of Health, resulted in the defeat of the Government by rine votes in a small House at a morning sitting. The old Board of Health was thus knocked on the head, a new Ministerial body replaced it and paved the way for variations in policy, while Chadwick, at the age of fifty-four, was relegated to private life on a pension of £1,000 a year.

### The True Sanitary Gospel.

During the thirty-six years which remained to Chadwick after the closure of his official life, he pursued, with little evidence of resentment and with the same ardour, the cause of sanitary and social reform. Pamphlets, presidential and other addresses, reviews and critiques came from his pen at frequent intervals and these, with his voluminous official reports, cover a period of sixty years of continuous labour. Whatever the verdict of history may eventually be, he stands for solid achievement in the story of sanitary and social reform. Much that was at the time ridiculed as the vapouring of a visionary has become the veriest commonplace of daily sanitary administration. He and his school of thought taught nineteenth-century England a very salutary lesson. They believed that they found in external physical conditions, largely removable and mostly the product of ignorance or neglect, the proximate antecedents of certain infections called zymotic. These were the nemesis of sanitary shortcomings, the evil fruitage of transgression of hygienic law, and, in a sense, therefore, the penalty of moral misdoing. In practical application they sought to influence for good the environment of mankind, making the battleground with disease without rather than within the uninfected body, setting great store on the natural vigour of the healthy body itself as the best security against its successful invasion by inimical agencies.

## Figures and Tables

**Figure f1:**